# Evidence summary on pain management in thoracoscopic lung cancer surgery

**DOI:** 10.1016/j.apjon.2025.100693

**Published:** 2025-03-24

**Authors:** Dongdong Wu, Jianjuan Dai, Yifan Sheng, Yin Lin, Hong Ye, Donglin Wang, Lina Lu, Boer Yan

**Affiliations:** a Department of Geriatric and Integrated Chinese and Western Medicine, Zhoushan Hospital, Zhoushan, China; b Department of Cardiothoracic Surgery, Zhoushan Hospital, Zhoushan, China; c Intensive Care Unit, Zhoushan Hospital, Zhoushan, China; d Department of Orthopedics, Zhoushan Hospital, Zhoushan, China; e Department of Gastrointestinal Surgery, Zhoushan Hospital, Zhoushan, China; f Department of Nursing, Zhoushan Hospital, Zhoushan, China

**Keywords:** Adult, Lung cancer, Thoracoscopic surgery, Pain management, Evidence summary

## Abstract

**Objective:**

The study aimed to systematically retrieve, evaluate and summarize evidence on perioperative pain management in adults undergoing thoracoscopic lung cancer surgery, to assist oncology nurses in improving pain assessment and management.

**Methods:**

The research question was established using PIPOST model and a systematic search was conducted in English and Chinese databases, professional society websites and guideline platforms for literature published between January 2017 and December 2024. Included literature types comprised guidelines, systematic reviews, evidence summaries, expert consensus, and standards. After literature searching and screening in January 2025, the remaining guidelines were evaluated by four investigators, while other literature was assessed by two investigators. Evidence was then extracted and graded.

**Results:**

Eighteen articles were included, comprising 5 systematic reviews, 3 guidelines, 2 clinical decisions, 4 evidence summaries, 3 expert consensus, and 1 standard. Twenty-five pieces of evidence across six topics were summarized, covering organizational management, high-risk patient assessment and preoperative education, pain assessment, intraoperative analgesia, multimodal pharmacological strategies, and non-pharmacological interventions.

**Conclusions:**

This evidence summary highlights effective strategies for perioperative pain management in thoracoscopic lung cancer surgery, which could support oncology nurses in implementing comprehensive pain assessment, identifying high-risk patients, and applying diversified analgesic interventions.

## Introduction

Surgical resection is the preferred treatment for stage I, II, and IIIA non-small cell lung cancer.^1^ Compared with traditional thoracotomy, thoracoscopic lung cancer surgery, as a minimally invasive thoracic surgery, has many advantages, such as reducing surgical trauma stress, enhancing postoperative rapid recovery, and decreasing the incidence of postoperative complications.^2^ However, even so, more than 50% of patients undergoing thoracoscopic lung cancer surgery still experience moderate to severe acute postoperative pain.^3^^,^^4^ A patient-reported outcome study regarding thoracoscopic surgery showed that pain is one of the five main symptoms for postoperative thoracoscopic surgery patients.^5^ If the pain is not well controlled, it will affect early postoperative activities and respiratory function exercises, leading to increased risk of postoperative complications, decreased patient satisfaction, prolonged hospital stay and extended duration of drainage tube placement.^6^, ^7^, ^8^ What is worse, the pain may even develop into chronic postoperative pain (CPSP).^9^ According to the International Association for the Study of Pain (IPSP), CPSP is pain that starts or becomes more intense after a surgical procedure or tissue injury and persists for 3 months after it.^10^ And CPSP can result in psychological distress and disability, thus affecting the long-term quality of life of patients.^11^ Studies have shown that the incidence of CPSP caused by thoracoscopic lung cancer surgery is as high as 43.9%.^12^ Thus, it can be seen that pain management for thoracoscopic lung cancer is critical.

Currently, nurses in our country are primarily responsible for pain assessment, monitoring treatment efficacy and side effects, and implementing non-pharmacological analgesia. In China, a consensus^13^ on thoracoscopic surgery pain management provides limited clinical guidance and has not been updated. Internationally, the PROSPECT guidelines^14^ is the most authoritative resource for perioperative pain management in thoracoscopic lung cancer, focusing on regional analgesia techniques and medications for anesthesiologists and surgeons. Nevertheless, pain management is multidisciplinary, and nurses play a crucial, irreplaceable role in this process.

Therefore, the study aimed to retrieve, evaluate and comprehensively summarize the pertinent evidence regarding perioperative pain management in adult individuals undergoing thoracoscopic lung cancer surgery. The summary will encompass aspects such as pain assessment during the thoracoscopic perioperative period, high-risk patient management, diverse pharmacological and non-pharmacological analgesia strategies, and intraoperative injury-control approaches. Traditionally, the perioperative period is defined as the period from 5 to 7 days preoperatively to 7–12 days postoperatively, whereas in this study it is expanded to include the entire process from preoperative evaluation to full postoperative recovery, highlighting the whole process of pain management.^15^ Hopefully, it can assist oncology nurses in better implementing pain assessment and management. This study has obtained registration on the platform of the Fudan University Center for Evidence-Based Nursing (Fudan CEBN, Registration No. ES20244369).

## Methods

This evidence summary was in line with the standards of the evidence summary report of Fudan CEBN, covering aspects such as problem formulation, evidence retrieval, inclusion and exclusion criteria of evidence, literature screening, literature quality evaluation, evidences extraction and summary.

### Problem formulation

The research question in this study was formulated based on the PIPOST model.^16^ To be specific, the first P (the targeted population) referred to adult lung cancer patients undergoing thoracoscopic pulmonary resection; I (intervention) represented pain assessment, pain treatment, and relevant education and management; the second P (the professionals who apply evidence) included anesthesiologists, thoracic surgeons, nurses, rehabilitation therapists and so on; O (outcome) covered the prevalence of moderate to severe pain during hospitalization, satisfaction with pain control, the effect of pain on patients' functional exercise, incidence of pain treatment-related complications and so on; S (the evidence-application setting) included the operation room, thoracic surgery wards and patients’ homes; T (type of evidence) consisted of system reviews, guidelines, expert consensus, standards and evidence summaries. Literature not published in English or Chinese was excluded.

### Evidence retrieval

Literature was searched according to the “6S” evidence resource pyramid model,^17^ and the search was carried out in order from top to bottom based on the level of evidence. The searched clinical decision support systems included UpToDate, BMJ Best Clinical Practice Network, and DynaMed. The guideline websites included Guidelines International Network (GIN), Scottish Intercollegiate Guidelines Network (SIGN), the website of the National Institute for Health and Care Excellence (NICE), Registered Nurses Association of Ontario (RNAO), World Health Organization (WHO), New Zealand Guidelines Group (NZGG), and Medlive. The professional society websites included American Pain Society (APS), American Society for Pain Management Nursing (ASPMN), American Society of Regional Anaesthesia and Pain Medicine (ASRA), American Society of Anaesthesia (ASA), Canadian Pain Society (CPS), European Society of Anaesthesiology (ESA), French Society for Anaesthesia and Intensive Care (SFAR), Society for Perioperative Assessment and Quality Improvement (SPAQI) and the Joint Commission. The comprehensive databases included Cochrane Library, Joanna Briggs Institute (JBI) Library, PubMed, Web of Science, Cumulative Index to Nursing and Allied Health Literature (CINAHL), Embase, PsycINFO, ClinicalKey, China National Knowledge Infrastructure (CNKI), VIP Database, Wanfang Database, and Chinese Medical Association.

**Fig. 1:**
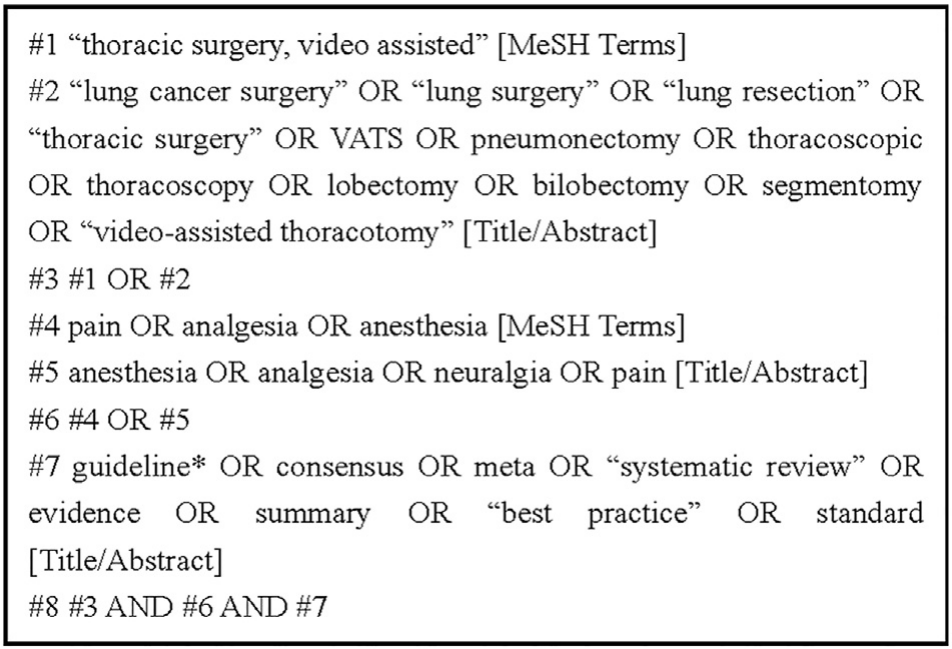
PubMed search strategy. VATS, video-assisted thoracoscopic surgery.

The terms “thoracoscopic surgery”, “pain”, “lung cancer”, and “anesthesia” were used to search clinical decision support systems, guideline websites, and professional society websites. The search in comprehensive databases was carried out in the form of Boolean logic operation combined with MeSH subject terms and free terms. The retrieval time limit was from January 2017 to December 2024. In addition to the above databases, relevant references were also traced back. An instance of searching strategy in PubMed is demonstrated in Fig. 1.

### Inclusion and exclusion criteria of evidence

The inclusion criteria were (1) the research subjects are adult lung cancer patients who have undergone video-assisted thoracoscopic lung resection; (2) the research content involves pain assessment, preventive analgesia, intraoperative and postoperative analgesia, pain quality control and so on; (3) the types of evidence include clinical decisions, practice guidelines, standards, systematic reviews, expert consensus and evidence summaries; (4) the language of the included literature is Chinese or English.

Exclusion criteria were (1) incomplete information; (2) publications that were duplicates; (3) abstracts, translations, interpretations, or guidelines that were out of date; (4) full-text articles that cannot be obtained; (5) low-quality evaluation levels.

### Literature screening

Initially, the retrieved literature was imported into EndNote to remove duplicates. Two researchers with a background in evidence-based nursing independently conducted the literature screening. In the first round of screening, they examined titles, abstracts and keywords. Subsequently, they read the full texts for re-screening and conducted a quality evaluation of the re-screened literature. Whenever there was a controversy over the inclusion of a retrieved document during the evaluation, a third expert in the core area of evidence-based nursing was consulted to resolve the issue and decide on its inclusion.

### Literature quality evaluation

The Appraisal of Guidelines for Research and Evaluation II (AGREE II) was utilized to appraise the guidelines.^18^ The standardized scores were categorized into three grades. For grade A, when the scores of all 6 items reached 60% or higher, the guideline was recommended as such. In the case of grade B, if the scores of 3 items were at least 30% and some scores were less than 60%, it was recommended after modification and improvement. As for grade C, areas with scores of 3 items less than 30% were excluded. The evaluation of systematic reviews made use of the revised version of the 2017-introduced systematic review tool, namely the Assessment of Multiple Systematic Review 2 (AMSTAR 2).^19^ The evaluation of expert consensus and standards used the JBI Evidence-based Health care Centre Text Evidence Quality Appraisal Checklist (2019 version).^20^ Clinical decisions and evidence summaries are positioned at the apex of the evidence resource pyramid, representing a high level of evidence. Currently, no corresponding tools exist to evaluate the quality of clinical decisions and evidence summaries. Therefore, the quality of these types of evidence was evaluated by tracing back to the original documents of each evidence source and choosing appropriate evaluation tools. The Newcastle–Ottawa Scale (NOS) was utilized for prospective or retrospective observational studies.^21^ And for Random Controlled Trials (RCTs), the Cochrane Handbook 5.1 version was adopted for quality evaluation.^22^

Four evidence-based-nursing-trained researchers independently undertook the quality evaluation of guidelines. Meanwhile, two other researchers were responsible for independently evaluating the quality of the remaining literature. In the event of disagreements during the evaluation process, an additional researcher was invited to contribute. To measure the inter-researchers consistency, the Intra-group Correlation Coefficient (ICC) was employed. When contradictions emerged among the evidence conclusions, preference was given to the highest quality evidence, most recently published and sourced from authoritative journals.

### Evidence extraction and summary

Evidence extraction and summarization were performed by two researchers trained in evidence-based nursing and experienced in thoracic surgery, following the principles below: (1) when the content of different pieces of evidence is consistent, the one with a more concise and professional expression is chosen. For example, in the context of multimodal analgesia, “Minimizing opioids is an important component of the multimodal analgesic strategies”^23^ and “There is currently an emphasis on multimodal pain control to minimize opioid pain medication when possible during perioperative care”,^24^ the latter statement was selected. (2) when the content from multiple sources is either similar or complementary, it is combined into a single piece of evidence. (3) when the content from multiple sources conflicts, the evidence with the highest quality or the one recently published in an authoritative evidence-based journal is included. Take nerve block in video-assisted thoracic surgery as an example. UpToDate proposed “Regional anesthetic techniques (e.g., epidural, paravertebral, or serratus anterior plane blocks) and nonopioid analgesics (e.g., acetaminophen, nonsteroidal antiinflammatory agents) are employed to minimize postoperative opioid use.”^23^ and its source is the enhanced recovery after lung surgery guideline published in the European Journal of Cardio-Thoracic Surgery by the European Society of Thoracic Surgeons in 2019.^25^ However, the guidelines for pain management in video-assisted thoracic surgery published in Anaesthesia by the European Society of Regional Anaesthesia and Pain Therapy in 2021 recommended “Regional analgesic techniques such as paravertebral block and erector spinae plane block are recommended. Serratus anterior plane block can be used as a second choice. Thoracic epidural analgesia is not recommended for postoperative analgesia.”^14^ Based on the principle of favoring the most current and credible source, the latter was selected. Both the evidence classification and the recommendation level are determined according to the standards of the 2016 version of the JBI Evidence-Based Healthcare Centre.^26^ The evidence classification is divided into 5 levels considering feasibility, appropriateness, significance and effectiveness. The evidence hierarchy, ranging from level 1 (highest) to level 5 (lowest), includes recommendations classified as Grade A (strong) or Grade B (weak).

## Results

### Search results and general information

**Fig. 2:**
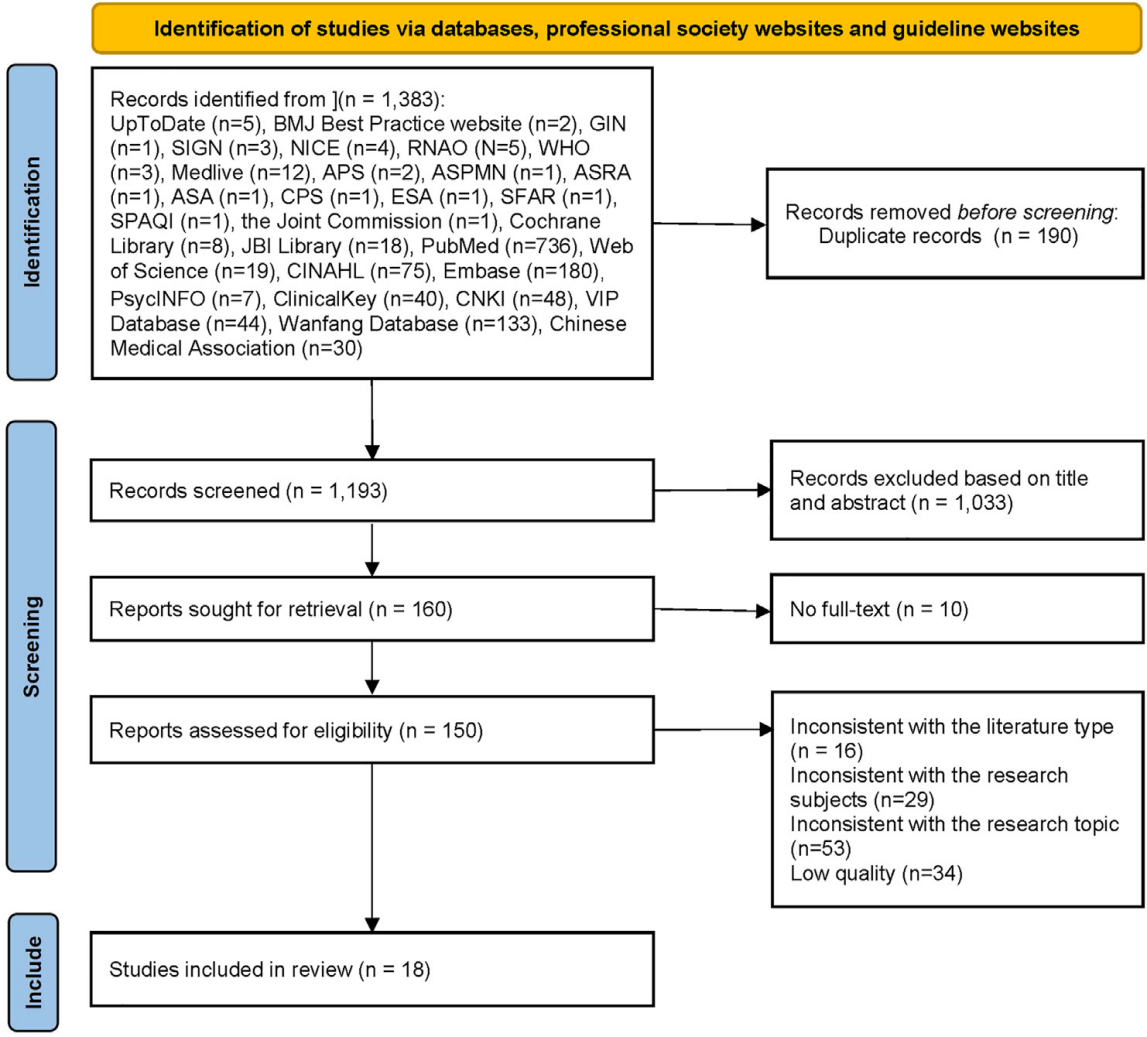
Flow chart of literature screening. GIN, Guidelines International Network; SIGN, Scottish Intercollegiate Guidelines Network; NICE, the website of the National Institute for Health and Care Excellence; RNAO, Association of Ontario; WHO, World Health Organization; APS, American Pain Society; ASPMN, American Society for Pain Management Nursing; ASRA, American Society of Regional Anaesthesia and Pain Medicine; ASA, American Society of Anaesthesia; CPS, Canadian Pain Society; ESA, European Society of Anaesthesiology; SFAR, French Society for Anaesthesia and Intensive Care; SPAQI, Society for Perioperative Assessment and Quality Improvement; JBI, Joanna Briggs Institute.

Finally, a total of 18 articles were included, consisting of 3 guidelines,^14^^,^^27^^,^^28^ 5 systematic reviews,^29^, ^30^, ^31^, ^32^, ^33^ 3 expert consensus,^13^^,^^24^^,^^34^ 2 clinical decisions,^23^^,^^35^ 4 evidence summaries,^36^, ^37^, ^38^, ^39^ and 1 standard.^40^ The literature screening process can be shown in Fig. 2, while the general characteristics of the included literature are presented in Table 1.

**Table 1:** Basic characteristics of the included literature.

Included literature	Publication time (year)	Type of evidence	Literature resource	Literature theme
Popescu et al.^23^	2023	UpToDate	UpToDate	Anesthetic management for enhanced recovery after thoracic surgery
Karamnov^35^	2023	UpToDate	UpToDate	Anaesthesia for thoracoscopic lung resection
Feray et al.^14^	2022	Guideline	PubMed	Pain management in thoracoscopic surgery
Aubrun et al.^27^	2019	Guideline	SFAR	Postoperative pain management
NICE^28^	2020	Guideline	NICE	Perioperative pain management for adults
Zhang et al.^32^	2023	Systematic review	Embase	Comparison of the effectiveness between uniportal and biportal thoracoscopic lobectomy
Zhang et al.^29^	2022	Systematic review	PubMed	Prevention of nausea and vomiting after thoracic surgery
Lim et al.^30^	2022	Systematic review	PubMed	Risk factors for chronic pain after thoracic surgery
Li et al.^31^	2022	Systematic review	PubMed	Perioperative outcomes of not routinely chest tube drainage after thoracoscopic pulmonary resection
Zhang et al.^33^	2021	Systematic review	Web of science	Comparison between non-intubated and spontaneous ventilation intubated thoracoscopic surgeries
Quinlan-colwell et al.^34^	2022	Expert consensus	ASPMN	The relationship between opioid dosage and pain intensity
O'Rourke et al.^24^	2021	Expert consensus	SPAQI	Preoperative medication management for patients who have been taking analgesic drugs for a long time
Zhu YK et al.^13^	2018	Expert consensus	Medlive	Perioperative pain management in thoracic surgery
The joint commission^40^	2017	Standard	The joint commission	Pain assessment and management
Valdez^36^	2023	Evidence summary	JBI	Postoperative pain management
Lizarondo et al.^37^	2023	Evidence summary	JBI	Pain assessment for surgical patients
Valdez^38^	2022	Evidence summary	JBI	Pain secondary to chest drain removal: Cold application
Moola^39^	2021	Evidence summary	JBI	Postoperative pain: massage

SFAR, French Society for Anaesthesia and Intensive Care; NICE, the website of the National Institute for Health and Care Excellence; ASPMN, American Society for Pain Management Nursing; SPAQI, Society for Perioperative Assessment and Quality Improvement; JBI, Joanna Briggs Institute.

### Quality evaluation results of literature

#### Quality evaluation results of guidelines

Three guidelines were included.^14^^,^^27^^,^^28^ Additionally, after tracing back to the original literature of clinical decisions and evidence summaries, three more guidelines were obtained.^25^^,^^41^^,^^42^ The ICC of the four researchers was all > 0.750, indicating a relatively high overall quality. These guidelines were approved for inclusion. In Table 2, the outcomes of the quality evaluation are displayed.

**Table 2:** Methodological evaluation results of guidelines (*N* ​= ​6).

Guidelines	Standardized scores in various domains (%)						≥ 60%	≥ 30%	ICC	Quality evaluation
	1. Scope and purpose	2. Stakeholder involvement	3. Rigor of development	4. Clarity of presentation	5. Applicability	6. Editorial independence				
Chou et al.^25^	98.61	98.61	83.85	94.44	66.67	83.00	6	6	0.821	A
Batchelor et al.^27^	94.44	76.39	80.21	97.22	45.83	92.00	5	6	0.768	B
Feray et al.^14^	70.83	54.17	58.33	70.83	55.21	75.00	3	6	0.761	B
Aubrun et al.^42^	77.78	70.83	67.71	86.11	48.96	65.00	5	6	0.764	B
NICE^28^	95.83	88.89	89.06	100.00	91.67	85.00	6	6	0.762	A
Schug et al.^41^	94.44	80.56	86.46	93.06	66.67	85.00	6	6	0.759	A

ICC, Intra-Class Correlation Coefficient; NICE, the website of the National Institute for Health and Care Excellence.

### Quality evaluation results of systematic reviews

Five systematic reviews included^29^, ^30^, ^31^, ^32^, ^33^ were evaluated by AMSTAR 2.^19^ In the studies conducted by Lim et al.,^30^ the evaluation result of item 2 was “partial yes”, and the results of the remaining items were “yes”. In the study carried out by Zhang et al.,^33^ the evaluation result of item 9 was “only include RCTs”, while the results of the rest of the items were “yes”. In the study of Zhang et al.,^32^ the results of items 2, 3 and 10 were “no”, the result of item 4 was “partial yes”, and the results of the remaining items were all “yes”. Regarding the studies by Zhang et al.^29^ and Li et al.,^31^ all items were evaluated as “yes”. The research designs of the aforementioned studies were relatively complete, so all of them were included.

### Quality evaluation results of expert consensus and standard

This study included three expert consensus.^13^^,^^24^^,^^34^ For the seven items evaluated in the expert consensus by O'Rourke et al.,^24^ all were rated as “yes”. For the expert consensus by Quinlan-Colwell et al. and Zhu YK et al.,^13^^,^^34^ items 4 and 5 were rated as “unclear”, and the remaining five items were rated as “yes”. The research design was reasonable and of high quality, so they were included. One standard was included.^40^ Item 4 was rated as “unclear”, and the remaining six items received “yes”. The overall quality was high, so it was included.

### Quality evaluation results of clinical decisions

We included two clinical decisions.^23^^,^^35^ After tracing back to the original literature of these evidence sources, we retrieved one guideline and two observational studies.^25^^,^^43^^,^^44^ The results of the quality evaluation of the guideline can be seen in Table 2.^23^ When evaluating the two observational studies with the NOS, both were found to be of high quality.^43^^,^^44^ Consequently, they were included.

### Quality evaluation results of evidence summaries

We included four evidence summaries.^36^, ^37^, ^38^, ^39^ After tracing the original literature of the evidence sources, two guidelines,^41^^,^^42^ two systematic reviews^45^^,^^46^ and one RCT^47^ were obtained. The quality evaluation of the two guidelines^41^^,^^42^ is also shown in Table 2. The quality of the systematic reviews^45^^,^^46^ was evaluated by AMSTAR 2.^19^ In the study by Engwall et al.,^45^ the results of items 2, 5, 6, 10, 13 and 16 were “no”, the results of items 11, 12 and 15 were “meta-analysis was not performed”, while items 1, 3, 4, 7, 8, 9, and 14 received “yes” as their results. Regarding the study by Kukimoto et al.,^46^ items 2, 10 and 16 had “no” as their results, item 9 had a result of “only include RCTs”, while the remaining items all obtained a “yes” result. Overall, the quality of these two studies was found eligible. The quality of one RCT^47^ was evaluated using the Cochrane Handbook 5.1. Except for item 3 “Blinding of all study participants and personnel (performance bias)” which was at high risk of bias, the remaining six items were at low risk of bias. The overall quality evaluation grade was B, so it was included.

### Description and summary of evidence

After extracting and integrating the evidence, a total of 25 pieces of evidence were ultimately formed, of which 17 were recommended at level A and 8 at level B (Table 3).

**Table 3:** Summary of the best evidence for the whole-process management of pain in thoracoscopic surgery for lung cancer in adults.

Category	Evidence content	Level of evidence	Level of recommendation
Organizational management	1. Hospitals should have an appropriate pain management framework to develop and improve safe and effective perioperative pain control policies and procedures, and conduct quality supervision and improvement.^40^	Level 5	A
	2. Evaluate the patient's physiological and mental comorbidities, concurrent medications, chronic pain and medication history, substance abuse, previous postoperative analgesia treatment plans and responses.^24^^,^^27^^,^^37^ develop a perioperative analgesia plan by integrating the patient's preferences, expectations and pain management goals.^30^^,^^40^	Level 5	A
	3. Regularly analyze pain assessment and management data, monitor the sedation level, occurrence of respiratory depression and other adverse events in patients using opioids, as well as the duration and dosage of opioid prescriptions.^38^ for other types of drugs involved in the multimodal analgesia regimen, their adverse reactions also need to be monitored.^13^	Level 5	A
Assessment of high-risk patients and preoperative education	4. Patients who are opioid-tolerant or have a history of substance abuse are at high risk of poor control of acute postoperative pain. It is advisable to request a consultation with a pain specialist or anesthesiologist.^13^	Level 5	B
	5. Patients suffering from sleep apnea, individuals on continuous intravenous opioid treatment, and those undergoing oxygen therapy are at high risk of adverse reactions to opioid analgesics. Devices for monitoring the adverse consequences of opioid therapy should be used during perioperative analgesia with opioids.^40^	Level 5	A
	6. Younger age, being female, having hypertension, experiencing preoperative pain, suffering from moderate to severe acute postoperative pain, the surgical approach adopted, the extent of surgical resection, and incision-related complications are all high-risk determinants for chronic pain following thoracic surgery,^30^ which should be given special attention.	Level 4	A
	7. Regarding patients who have taken analgesic medications over a long time, the preoperative pain control status should be evaluated. When formulating a perioperative drug-based analgesia plan, the drug interactions and the nature of the medications should be taken into consideration to decide whether to discontinue the medications.^24^	Level 5	B
	8. Carry out comprehensive pain education before surgery. The specific content includes emphasizing to patients and their families the significance of early extubation, relieving postoperative pain, accelerating recovery, and shortening the hospital stay,^23^ so as to reduce the use of opioid drugs and better control postoperative pain.^13^	Level 5	A
Pain assessment	9. Select an appropriate pain assessment tool based on the patient's cognition, consciousness, educational level, and language, and modify the analgesia plan appropriately according to the assessment results.^37^	Level 5	A
	10. Considering the patient's age, state of health, and comprehension ability, define the criteria for screening and reassessing pain. The time for re-evaluating pain after treatment should be the time when the blood concentration of the analgesic reaches its peak.^37^^,^^40^	Level 5	A
	11. The content of pain assessment should include the pain location, intensity, type, impact on functional activities, barriers to effective pain management, the patient's psychological status, opioid tolerance, and whether a new medical condition or surgical complications may cause the postoperative pain. Among them, the pain locations after thoracoscopic surgery involve the incision site and the ipsilateral shoulder,^35^ and both static and dynamic pain should be evaluated simultaneously.	Level 5	A
Intraoperative analgesia	12. During thoracoscopic surgery, paravertebral or erector spinae plane block is the top-choice regional analgesia technique, whereas the serratus anterior plane block is the secondary selection. Thoracic epidural analgesia or intrapleural local anesthetic analgesia is not recommended.^14^	Level 1	A
	13. Position the patient in a lateral posture on the non-affected side. Angle the operating table downwards to maximize the opening of the intercostal space. This approach aims to reduce the incision pain caused by the surgical instruments compressing the intercostal nerve.^35^	Level 5	B
	14. When the patient's condition and treatment permit, giving priority to not placing a thoracic drainage tube,^31^ performing single-port laparoscopic surgery,^32^ and non-intubated anaesthesia to alleviate the extent of acute postoperative pain through intraoperative damage control.^33^	Level 1	B
Multimodal pharmacological strategies	15. Paracetamol or nonsteroidal anti-inflammatory drugs should be administered as prophylactic analgesic agents before surgery, and continue their use postoperatively.^14^	Level 1	A
	16. 20 minutes before the end of the surgery, dexmedetomidine should be intravenously infused to curtail the utilization of postoperative opioids as well as the occurrence rate of postoperative nausea and vomiting, especially in scenarios where prophylactic analgesia and regional analgesia techniques are unavailable.^14^^,^^28^	Level 1	A
	17. In patients who are at high risk of poor control of acute postoperative pain or chronic postoperative pain, especially those with a long-term history of opioid use or opioid addiction, a low dose of ketamine (ranging from 0.125 to 0.25 mg/kg/h, with a maximum not exceeding 0.5 mg/kg/h) can be used after anaesthesia induction to reduce the pain intensity during the initial 24 hours post-surgery and decrease the incidence of chronic postoperative pain. It is crucial to halt the infusion 30 minutes prior to the conclusion of the surgery.^27^	Level 1	A
	18. Opioid analgesics are an important part of multimodal analgesia. Oral administration is the top-choice route. When patients cannot take medications orally, a patient-controlled intravenous analgesia pump is recommended.^27^	Level 1	B
	19. The determination of the dosage of opioid analgesics must be based on an individualized and holistic pain assessment. The practice of determining the dosage of opioid analgesics solely according to the patient's pain intensity is prohibited.^34^ regarding patients who face the risk of respiratory depression subsequent to thoracic surgery, such as the elderly or those with underlying pulmonary diseases, the use of opioids is restricted,^23^ but they should still be retained as postoperative rescue analgesics.^14^	Level 5	A
	20. For patients without contraindications, it is advisable to use non-opioid drugs such as paracetamol or nonsteroidal anti-inflammatory drugs, and gabapentin or pregabalin as part of multimodel analgesia to reduce the dosage of perioperative opioid use.^23^^,^^24^	Level 5	A
	21. Patients receiving out-of-hospital drug-based pain relief should be instructed on how to safely store and use opioid drugs, potential side effects of pain treatment, factors that may exacerbate or alleviate pain, how pain impacts daily life activities, and strategies to address these issues.^40^	Level 5	A
Non-pharmacological interventions	22. Nurses should be encouraged to use non-pharmacological interventions as supplementary measures to relieve postoperative pain. These specifically include patient education,^12^ communication,^21^ music therapy,^36^ massage,^39^ cold compress^38^ and so on.	Level 5	A
	23. Music therapy can be adopted. The specific method is to choose a quiet and relaxing environment. The types of music can include piano music, harp music, orchestral music, or slow-tempo jazz without lyrics. The music can be brought by the patient themselves or prepared by medical staff. It is recommended that each playing session lasts about 30 minutes.^36^	Level 5	B
	24. Massage can be used to promote relaxation and reduce postoperative pain. The area to be massaged is determined according to the location of the pain. Massage methods can include Swedish massage, comprehensive massage therapy, basic aromatherapy, or effleurage. It is recommended that each massage session last about 20 minutes.^39^	Level 5	B
	25. An ice pack can be applied at the catheter insertion site before the chest drainage tube is removed to relieve the secondary pain induced by the extraction of the closed thoracic drainage tube.^38^	Level 5	B

## Discussion

In this study, we searched different types of literature in multiple databases regarding pain assessment and management of thoracoscopic lung cancer surgery. We found that different literature had different focuses on the perioperative pain management of thoracoscopy, and there were conflicting recommendations among the literature. Therefore, synthesizing the evidence is essential to provide clear and feasible pain management recommendations for oncology nurses and other relevant medical professionals. The evidence developed in our study can be divided into six major themes: organizational management, high-risk patient assessment and preoperative education, pain assessment, intraoperative analgesia, multimodal pharmacological strategies, and non-pharmacological interventions.

### Establishing a pain organizational management system is the prerequisite for pain management in thoracoscopic lung cancer surgery

Evidence 1–3 indicates that hospitals should set up a pain organizational management system. Its function is to formulate and improve the policies and procedures related to perioperative pain management, develop perioperative analgesia plans based on patients' individual conditions, and conduct quality supervision and improvement during the implementation of analgesia treatment. Although the level of evidence is relatively low, it has guiding significance for clinical practice and should be promoted. Perioperative pain management involves multiple disciplines. Forming a multidisciplinary management team, including anesthesiologists, pain nurses, clinicians, and ward nurses, is conducive to the safe and effective clinical practice of pain management. It is recognized that establishing a pain organizational management system such as acute pain service (APS), contributes to the efficiency of pain management.^48^ Furthermore, previous literature^41^ illustrated that involving patients in medical decision-making can improve treatment outcomes. Therefore, when formulating perioperative analgesia plans, in addition to considering patients' coexisting physiological and mental disorders, concomitant medications, chronic pain status, medication histories, drug abuse records, and previous postoperative analgesia treatment plans and associated responses,^24^^,^^27^^,^^37^ patients’ expectations and preferences also need to be considered,^30^ and the adverse reactions of analgesic drugs should be monitored during the implementation of analgesia treatment.

### Implementing the assessment of high-risk patients is the guarantee for pain management in thoracoscopic lung cancer surgery

Evidence 4–8 summarizes the importance of assessing of high-risk patients, the preoperative management of patients with long been taking analgesic medications, and preoperative health education in pain management during the thoracoscopic perioperative period. The entire process management of pain throughout the thoracoscopic perioperative phase involves three types of high-risk patients, namely those at high risk of poor postoperative acute pain control,^13^^,^^41^ those at high risk of adverse reactions to opioid analgesics,^40^ and those facing the high risk of chronic pain following thoracic surgery.^30^ For high-risk patients, it is necessary to detect them promptly, intervene as early as possible, communicate with them fully, and track them throughout the process to ensure safe perioperative analgesia management. In addition, for patients with preoperative pain, good preoperative pain control is connected to the severity of postoperative pain and the consumption of opioid drugs. Clinical medical staff must pay attention to their pain control level and evaluate the interaction between drugs and the nature of drugs to decide whether to stop the drugs.^24^

### Individualized pain assessment is the basis of pain management in thoracoscopic lung cancer surgery

Evidence 9–11 summarizes the selection of pain assessment tools, the establishment of re-evaluation time and the specific content of pain assessment. Currently, commonly used pain assessment tools include the Numeric Rating Scale (NRS), Visual Analogue Scale (VAS), Verbal Rating Scale (VRS) and Faces Rating Scale (FRS) and so on.^37^ Selecting an appropriate pain assessment tool is a prerequisite for correct assessment, and correct pain assessment is a necessary condition for precise analgesia. The time for re-evaluating pain after treatment should be when the blood concentration of the analgesic reaches its peak.^37^^,^^40^ In the group standard “Adult Postoperative Pain Assessment and Nursing” of the Chinese Nursing Association,^49^ it is clearly stated that the re-evaluation time is 5–15 minutes after intravenous administration, 30 minutes after subcutaneous or intramuscular injection, and 1 hour after oral or rectal administration. These specific time intervals are also set based on the peak blood concentration of the drug, which is consistent with the evidence summary results in this article. In terms of pain site assessment, clinically, it is usually considered that the pain site of postoperative patients is at the incision, and it is easy to ignore the pain conditions at sites other than the incision. However, the incidence of ipsilateral shoulder pain after thoracoscopic surgery can be as high as 53%.^35^ It may be the main complaint of patients who have good control of incision pain through regional block. It usually occurs within 2 hours after surgery and lasts for 1–3 days or until the chest drainage tube is removed. The common cause of ipsilateral shoulder pain is that the diaphragm, pericardium and mediastinum pleural surfaces are stimulated and transmitted through the phrenic nerve. Shoulder ligament injury accompanied by myofascial involvement or main bronchus transection is a contributing factor. Ipsilateral shoulder pain can lead to chronic hyperesthesia, hypoesthesia, dysesthesia, allodynia and reduced activity. Based on the pain characteristics after thoracoscopic surgery, it is recommended that clinical medical staff pay attention to the pain conditions and their influencing factors at non-incision sites during postoperative assessment.

### Intraoperative analgesia is key to pain management in thoracoscopic lung cancer surgery

Evidence 12–14 points out how to perform intraoperative analgesia. Evidence 12 and 14 have a relatively high level of evidence and can be directly applied. Although the level of evidence of item 13 is relatively low, it is easy to implement and promote. Peripheral regional analgesia techniques have been proven to be a practical part of multimodal analgesia. They can be used in pain management during the thoracoscopic perioperative period. The analgesic effect of single-injection regional analgesia technique is definite. Moreover, when the duration of postoperative pain exceeds the expected analgesic duration of a single injection, continuous regional analgesia technique is preferably used, reflecting the continuity of analgesic treatment.^41^ The research results on the effectiveness of intrathecal analgesia with local anesthetics for postoperative pain after thoracoscopic surgery are inconsistent. Additionally, this administration method has a high systemic absorption and may lead to potential toxic reactions, so it is not recommended.^14^ Epidural anaesthesia has no advantage in analgesic effect compared with regional analgesia,^50^^,^^51^ but it increases the incidence of postoperative hypotension,^52^ so it is not recommended. Not placing a chest drainage tube, single-port endoscopic surgery and non-intubated anaesthesia are all intraoperative injury control measures,^32^^,^^33^ which can reduce the degree of postoperative acute pain and can be considered when the condition and treatment allow. There is no high-quality original research to support the positioning strategy of bending the operating table downward to open the patient's intercostal space as much as possible. However, using this positioning strategy to reduce the compression of the intercostal nerves by surgical instruments has theoretical significance, so it is recommended.^35^

### Multimodal pharmacological strategies are the core of pain management in thoracoscopic lung cancer surgery

Multimodal pharmacological strategies play a crucial role in the pain management of thoracoscopic lung cancer surgery. Evidence 15–21 outlines these strategies before, during and after surgery. Notably, items 15–18 have relatively high evidence levels and can be directly applied in clinical practice, while items 19–21 with low evidence levels, require adaptation according to actual clinical circumstances. The fundamental approach to perioperative pain management for thoracoscopic lung cancer is multimodal analgesia, which involves nerve block, intravenous analgesia pump, nonsteroidal anti-inflammatory drugs and more.^7^ However, in clinical settings, numerous challenges exist in thoracoscopic perioperative pain management. During preoperative pain assessment, health care providers must account for patients’ physiological and psychological differences. Balancing the depth of anaesthesia and associated complications, coordinating different analgesic drugs in postoperative analgesia, and monitoring and managing CPSP are also complex tasks. The absence of unified standards for these aspects renders thoracoscopic perioperative pain management a demanding endeavor.

According to the time course of postoperative pain occurrence, the 24 hours after surgery is the most painful period for patients undergoing thoracoscopic surgery.^53^ Under the condition of no contraindications, it is recommended to administer acetaminophen or nonsteroidal anti-inflammatory drugs as preventive analgesics before surgery to promote the blood concentration of analgesics in the body to reach a peak within the 24-h time window after surgery, improve the coincidence between the blood concentration of analgesics and the severity of pain, and achieve good analgesic effects.^14^ The drugs that can be used during surgery are dexmedetomidine and low-dose ketamine.^14^^,^^27^^,^^28^ The effect of dexmedetomidine in reducing the consumption of postoperative opioid drugs and the incidence of postoperative nausea and vomiting (PONV) has been verified, and it is especially suitable for surgical patients who cannot be given preventive analgesia and regional analgesia techniques. Low-dose ketamine is used for pain control in patients at high risk of poor postoperative acute pain control and patients at high risk of CPSP. It should be noted that when using ketamine, the dose must be strictly controlled and the infusion should be stopped 30 minutes before the end of the operation. Continuous use after surgery will increase neuropsychiatric toxic reactions such as auditory hallucinations and talking to oneself. In terms of postoperative drug treatment, the preferred route of administration of opioid drugs is oral. For those who cannot take it orally, patient-controlled intravenous analgesia (PCIA) is recommended.^27^ Intramuscular injection is not encouraged. Its analgesic effect shows no advantage over oral, intravenous and rectal administrations. Moreover, intramuscular injection itself can cause significant pain and the absorption of analgesics in the body is uneven. The dose of opioid analgesics needs to be determined based on individualized and comprehensive pain assessment. The practice of determining the dose of opioid analgesics only based on the patient's pain severity is prohibited,^34^ as this practice ignores the relevance of evaluating other basic elements and may lead to adverse outcomes such as over-sedation and respiratory depression in patients. Acetaminophen/ nonsteroidal anti-inflammatory drugs and gabapentin/pregabalin can be used in combination for postoperative multimodal analgesia in patients without contraindications to reduce the consumption of opioid drugs throughout the perioperative phase.^23^^,^^24^ At the same time, continuous pain management services should be provided for patients with outpatient analgesic treatment.

### Non-pharmacological interventions are the supplement of pain management in thoracoscopic lung cancer surgery

Evidence 22–25 summarizes the non-pharmacological interventions within the scope of nursing. Among them, the evidence level of item 22 is relatively high, and the evidence levels of items 23, 24 and 25 are relatively low. However, the recommended massage therapy is easy to implement, popularize and has clinical application value. The mechanism of music therapy is the gate control theory, which holds that the mechanism in the dorsal horn of the spinal cord is like a gate that inhibits or promotes the transmission of pain impulses from the body to the brain. This gate is influenced by cognitive and emotional factors.^36^ Beautiful music exerts an analgesic effect by improving patients' cognition and regulating emotions. The mechanism of massage analgesia is to relieve local muscle spasms, improve blood circulation and relieve fatigue through mechanical stimulation. The principle of cold compress analgesia is to inhibit the conduction of pain nerves and increase the pain threshold.^38^ Previous studies have shown that nurses who use nonpharmacological interventions such as massage and transcutaneous electrical nerve stimulation (TENS) therapy reduce patients' postoperative pain and decrease analgesics use.^54^, ^55^, ^56^ Although the effectiveness of these non-pharmacological interventions in analgesic treatment is limited, they are highly operable and safe. They can be used as an adjuvant means of multimodal analgesia and can reduce the demand for opioid drugs to a certain extent, thus reducing the side effects of opioid drugs. When selecting non-pharmacological adjuvant analgesic measures, they can be determined according to patients’ preferences.

### Relevance to clinical practice

This study systematically summarizes the evidence-based foundation for the whole-process management of pain during thoracoscopic perioperative period, providing a reference for clinical practice. Compared with previous literature, this study emphasizes the following key points:

First, it identifies and manages three subgroups of patients at high risk of pain during the perioperative period of thoracoscopy. Specifically, these include patients at high risk of poor perioperative analgesia, patients at high risk of adverse reactions to opioid use, and patients at high risk of chronic pain after thoracoscopy.

Second, it defines the common pain sites after thoracoscopy, namely the surgical incision area and the ipsilateral shoulder. This precise positioning provides a basis for targeted pain assessment and the formulation of treatment strategies.

Third, it analyzes a series of detailed key points in the pain assessment and multimodal analgesia treatment during the perioperative period of thoracoscopy. Specifically, by basing on the peak blood-drug concentration time of analgesic drugs, health care providers plan the time point for re-evaluation after drug administration to ensure the timeliness and accuracy of pain assessment. During the operation, health care providers, by designing the body-position placement plan, reduce the compression of the intercostal nerves by surgical instruments, thereby alleviating the degree of postoperative incision pain. For patients using opioids for the first time, when health care providers administer drugs through an intravenous analgesia pump, they follow the principle of not setting a background dose to reduce the risk of adverse reactions to opioids.

Fourth, it elaborates on the specific implementation paths of non-pharmacological adjuvant analgesia measures within the scope of nursing care, such as music therapy, cold compress, and massage.

### Limitations and future research direction

Despite the comprehensive nature of this evidence summary on the pain management throughout thoracoscopic surgery for adult lung cancer patients, several limitations must be acknowledged. Geographical, racial, and cultural variances can introduce confounding factors that may influence the generalizability of the research findings. Additionally, the study's scope was restricted to Chinese and English databases, potentially overlooking valuable literature published in other languages. The omission of gray literature also indicates an area for refinement in the literature search methodology. Moreover, when translating this evidence into clinical practice, the diverse health care contexts necessitate careful consideration of the feasibility, suitability, and effectiveness of the proposed strategies.

For future research, our analysis has revealed a dearth of high-quality randomized trials examining non-pharmacological interventions post-thoracoscopic surgery within the purview of nursing practice. This gap in evidence limits the robustness and applicability of the current knowledge base. Regarding CPSP management following thoracoscopic surgery, existing studies predominantly concentrate on the incidence of CPSP rather than its treatment. Future scholars are thus encouraged to direct their efforts towards filling these critical research gaps.

## Conclusions

This evidence summary, grounded in current research, provides oncology nurses with valuable insights for managing pain in lung cancer patients undergoing thoracoscopic surgery. By integrating comprehensive pain assessment, high-risk patient identification, diversified analgesic interventions, and perioperative pain education, nurses can enhance patient care. However, when applying it to practice, it is important to analyse both facilitators and barriers to determine suitable approach. Future research should focus more on high-quality randomized trials within the nursing scope, particularly in perioperative pain management and addressing CPSP. Regular updates to this evidence summary are essential to ensure its relevance and accuracy in guiding clinical practice.

## CRediT authorship contribution statement

Dongdong Wu: Conceptualization, Methodology, Writing – Original Draft. Jianjuan Dai, Yifan Sheng, Yin Lin, Donglin Wang: Data Curation, Formal Analysis. Lina Lu, Hong Ye: Investigation. Boer Yan**:** Resources, Supervision, Writing – Review & Editing. All authors have read and approved the final manuscript.

## Ethics statement

Not required.

## Data availability statement

The data supporting the results of this study can be obtained from the corresponding author, BY, upon reasonable request.

## Declaration of generative AI and AI-assisted technologies in the writing process

No AI tools/services were used during the preparation of this work.

## Funding

This work was supported by the Medical Science and Technology Project of Zhoushan Municipal Health Commission (Grant No. 2022YB10).

## Declaration of competing interest

The authors declare no conflict of interest.
